# Identification of c-di-GMP Signaling Components in *Xanthomonas oryzae* and Their Orthologs in Xanthomonads Involved in Regulation of Bacterial Virulence Expression

**DOI:** 10.3389/fmicb.2019.01402

**Published:** 2019-07-11

**Authors:** Fenghuan Yang, Dingrong Xue, Fang Tian, William Hutchins, Ching-Hong Yang, Chenyang He

**Affiliations:** ^1^State Key Laboratory for Biology of Plant Diseases and Insect Pests, Institute of Plant Protection, Chinese Academy of Agricultural Sciences, Beijing, China; ^2^Department of Biology, Carthage College, Kenosha, WI, United States; ^3^Department of Biological Sciences, University of Wisconsin-Milwaukee, Milwaukee, WI, United States

**Keywords:** c-di-GMP signaling, regulation, virulence, pathogenesis, Xanthomonads

## Abstract

*Xanthomonas oryzae* pv. *oryzae* (*Xoo*) causes bacterial leaf blight of rice, one of the most devastating bacterial diseases of this staple crop worldwide. *Xoo* produces a range of virulence-related factors to facilitate its pathogenesis in rice, however, the regulatory mechanisms of *Xoo* virulence expression have been not fully elucidated. Recent studies have revealed that virulence factor production is regulated via cyclic dimeric guanosine monophosphate (c-di-GMP) signaling pathway that is well-conserved in *Xoo* and other *Xanthomonas* species. A set of GGDEF, EAL, HD-GYP, and PilZ domain proteins with diverse signal sensory domains for c-di-GMP synthesis, hydrolysis, and binding is encoded in the *Xoo* genome. Bioinformatic, genetic, and biochemical analysis has identified an array of diguanylate cyclases (DGCs) and phosphodiesterases (PDEs), as well as degenerate GGDEF/EAL, PilZ domain proteins along with a transcription regulator. These signaling components have been characterized to regulate various bacterial cellular processes, such as virulence, exopolysaccharide (EPS) production, biofilm formation, motility, and adaptation at the transcriptional, post-translational, and protein-protein interaction levels. This review summarized the recent progress in understanding the importance and complexity of c-di-GMP signaling in regulating bacterial virulence expression, highlighting the identified key signal elements and orthologs found in Xanthomonads, discussing the diverse functions of GGDEF/EAL/HD-GYP domains, existence of a complicated multifactorial network between DGCs, PDEs, and effectors, and further exploration of the new c-di-GMP receptor domains. These findings and knowledge lay the groundwork for future experimentation to further elucidate c-di-GMP regulatory circuits involved in regulation of bacterial pathogenesis.

## Introduction

The Gram-negative plant pathogen *Xanthomonas oryzae* pv. *oryzae* (**Xoo**) causes bacterial leaf blight disease of rice, one of the most devastating bacterial diseases of this staple crop in the world, leading to yield losses of rice production up to 20–50% under disease-favorable conditions (Mew et al., 1993; Niño-Liu et al., 2006). *Xoo* has become an ideal model to study the molecular mechanisms of bacterial pathogenesis in monocot plants (Niño-Liu et al., 2006; He et al., 2007; Salzberg et al., 2008; White and Yang, 2009). Typically, *Xoo* infects rice leaves through wounds or hydathodes, and then invades xylem vessels leading to systemic infection (Mew et al., 1993). Like many other Gram-negative bacteria, *Xoo* also possesses a type III secretion system (T3SS) to inject and deliver effectors into rice cells, some of which are critical determinants of pathogenesis during *Xoo*–rice interaction (Tsuge et al., 2006, 2014; White and Yang, 2009). Furthermore, diverse regulators including transcriptional factors, two-component transduction systems (TCS), and diffusible signal factor (DSF) pathways control the expression of virulence factors in a cooperative manner and contribute significantly to the virulence of *Xoo* *in planta* (Jha et al., 2007; Das et al., 2009; White and Yang, 2009; He et al., 2010; Yang et al., 2012; Tian et al., 2015; Li H. Y. et al., 2018). However, the regulatory mechanisms of bacterial virulence expression have been not fully understood in *Xoo*.

Pathogenicity of *Xoo* was immediately initiated in rice-*Xoo* interactions, while the expressions of T3SS-related *hrp* and c-di-GMP metabolic enzyme and receptor genes were significantly changed. For example, *hrpG* and *hrpX* were upregulated in 5 min after interacting with rice leaves, while 5 genes encoding c-di-GMP metabolic enzymes were firstly down-regulated, and then up-regulated. Absolute expression level of c-di-GMP receptor Clp was high, but its fold changes in expression were low, suggesting that Clp activity might be transcriptionally and/or post-translationally controlled via its binding to c-di-GMP. These studies indicate that both *hrp*-encoded T3SS and c-di-GMP signaling pathways play the important roles in interaction of *Xoo* to host rice (Kim et al., 2016). *Xoo* strains PXO99 and PXO86 from Philippines and GD1358 from China exhibited different virulence patterns in 30 rice varieties. Transcriptional expression profiling demonstrated that expression patterns of virulence-related genes including *avrXA21, hrpG* and the *gum* cluster in GD1358 were differential from those in PXO99 and PXO86. Thus, *Xoo* strains from the different regions exhibited distinctly different expression patterns of putative virulence-related genes (Zhang F. et al., 2013). A range of additional virulence-related factors, including extracellular polysaccharide (EPS) synthesis, biofilm formation, motility, extracellular cellulase, xylanase, catalase, and adhesin, are produced by *Xoo* to facilitate its pathogenesis (Ray et al., 2000; Jha et al., 2007; Das et al., 2009; White and Yang, 2009). Further insight into signaling pathways involved in regulation of virulence will contribute to the understanding of *Xoo* pathogenesis, and effectively allow the development of prevention strategies to facilitate new methods of control of pathogenic infection in rice.

Cyclic dimeric guanosine monophosphate (c-di-GMP) is a widespread second messenger in pathogenic bacteria (Hengge, 2009). The importance of c-di-GMP involvement in the regulation of various biological processes, such as virulence, EPS production, biofilm formation, motility, and pathogen environment adaptation has been illustrated in several bacteria (Hengge, 2009, 2013; Jenal et al., 2017). Downstream effects of c-di-GMP are dose-dependent; lower concentrations of intracellular c-di-GMP promote virulence factor production and motility, while higher concentrations invariably facilitate biofilm formation in bacteria (Jenal et al., 2017). The opposing actions of diguanylate cyclase (DGC) and phosphodiesterase (PDE) enzymes control the synthesis and degradation of c-di-GMP, respectively (Schirmer and Jenal, 2009). Generally, DGCs contain a conserved GGDEF domain, while PDEs possess conserved EAL or HD-GYP domains (Dow et al., 2006; Schirmer, 2016; Römling et al., 2017). Besides containing GGDEF, EAL, or HD-GYP domains, these proteins possess additional signal sensory and recognition domains that can integrate diverse environmental signals to the c-di-GMP signaling pathway (Römling et al., 2013; Jenal et al., 2017).

c-di-GMP binding promotes changes in the structure, as well as the function of the receptor/effector. This interaction allows changes in gene expression or enzyme activity to occur (Chou and Galperin, 2016; Yang et al., 2017). Several c-di-GMP receptors have been characterized from various bacterial species, including PilZ domain proteins, transcriptional regulators, degenerate GGDEF or EAL domain proteins, polynucleotide phosphorylase (PNPase), riboswitches and kinases (Hengge, 2009, 2013; Wang et al., 2012; Römling et al., 2013; Chou and Galperin, 2016). Functionally, c-di-GMP receptors exert their regulatory functions at the transcriptional, translational, and post-translational levels (Hengge, 2009, 2013; Jenal et al., 2017). Identification of receptors/effectors is of scientific importance, providing the basis for elucidation of c-di-GMP signaling pathway regulation of downstream functions.

Due to the economic importance of bacterial blight of rice and the excellent model of bacterial pathogens in monocot plants, recent studies on *Xoo* and close *Xanthomonas* species have revealed the importance and complexity of c-di-GMP signaling pathways in regulating virulence. The array of key components involved in c-di-GMP signal metabolism, reception, transduction and phenotypic regulation has been functionally identified and characterized, and regulatory circuits of virulence at the transcriptional, post-translational, and protein-protein interaction levels have been demonstrated. This review summarized the recent progress in understanding c-di-GMP signaling pathways and regulation of virulence in *Xoo*, all while relating those findings to other close *Xanthomonas* spp. including *X*. *oryzae* pv. *oryzicola* (Xoc), *X*. *campestris* pv. *campestris* (Xcc), and *X*. *axonopodis* pv. *citri* (Xac), highlighting the identified key signal elements and orthologs, discussing diverse functions of GGDEF/EAL/HD-GYP domains, existence of a complicated multifactorial network between DGCs, PDEs and effectors, and further exploration of new c-di-GMP receptor domains. These findings and knowledge lay the groundwork for future experimentation to further elucidate c-di-GMP regulatory circuits involved in the regulation of bacterial pathogenesis.

## Recognition of the GGDEF, EAL and HD-GYP Domain Proteins

**Figure 1:**
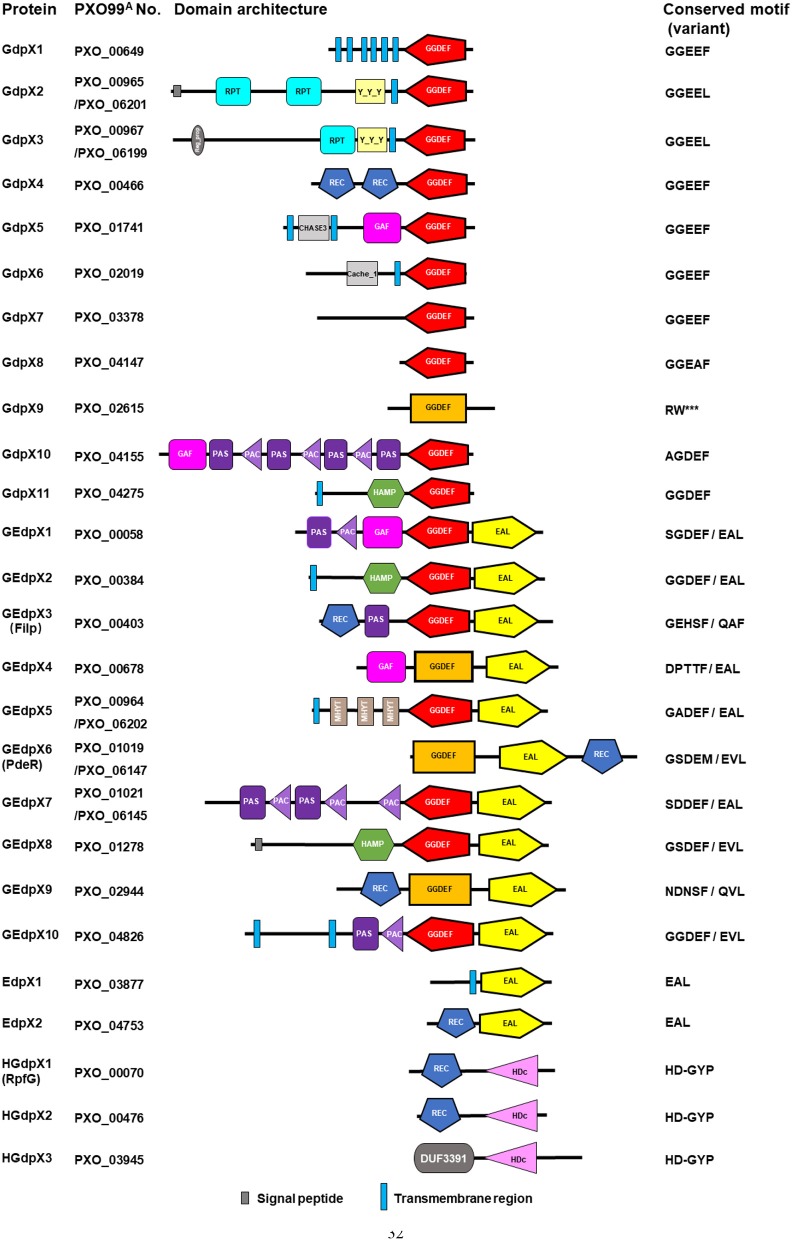
Identification of GGDEF, EAL and HD-GYP domain architecture and conserved motif in *Xanthomonas oryzae* pv. *oryzae*. Simple Modular Architecture Research Tool (SMART) (http://smart.embl-heidelberg.de/) was used to analyze the domains. The GGDEF domains implicated in c-di-GMP synthesis were shown in red, while the degenerate GGDEF domains in rectangle were predicted by SMART only with the Pfam database and displayed in orange. The EAL and HD-GYP domains implicated in c-di-GMP hydrolysis are illustrated in yellow and pink, respectively. ^***^means amino acid deficiency when compared with homologous proteins.

In the genome of *Xoo* PXO99^A^, 26 genes were annotated to encode GGDEF, EAL, and HD-GYP domain proteins revealed by bioinformatic analysis, including 11 *G*GDEF *d*omain standalone *p*roteins (GdpX1-11), 2 *E*AL *d*omain standalone *p*rotein (EdpX1-2), 10 *G*GDEF/*E*AL *d*omain *p*roteins (GEdpX1-10), and 3 *H*D-*G*YP *d*omain *p*roteins (HGdpX1-3) (Figure 1) (Yang et al., 2012; Xue et al., 2015). A 212-kb genomic duplication, including PXO_00964, PXO_00965, PXO_00967, PXO_01019 (*pdeR*), and PXO_01021 was identified in the genome of *Xoo* strain PXO99^A^ (Salzberg et al., 2008; Yang et al., 2012). Since having two copies in the genome, two designations PXO_01019 and PXO_06147 of *pdeR* were given accordingly (Table 1).

**Table 1:** Identification of c-di-GMP metabolizing enzymes, receptors, and effectors in *Xanthomonas oryzae* pv. *Oryzae*.

**Protein**	**PXO99^**A**^ number**	**Domain**	**Mode of action**	**Biological output**	**References**
**c-di-GMP METABOLIZING ENZYME**					
GdpX1	PXO_00649	GGDEF	DGC for c-di-GMP synthesis	Suppressing virulence, EPS production and motility	Yang et al., 2016
DgcA	XOO3988	GGDEF	DGC for c-di-GMP synthesis	Suppressing virulence, EPS production, motility, and bacterial autoaggregation; affected DGC and PDE expression	Su et al., 2016; Zhou H. et al., 2016
EdpX1	PXO_03877	EAL	PDE for c-di-GMP degradation	Promoting virulence and EPS production	Xue et al., 2018
VieAxoo	PXO_04753	EAL	PDE for c-di-GMP degradation	Promoting biofilm formation and induction of hypersensitive response in non-host	Liang et al., 2011
PdeR	PXO_01019/PXO_06147	GGDEF/EAL	PDE for c-di-GMP degradation	Promoting virulence and EPS production	Yang et al., 2012
RpfGxoo	PXO_00070	HD-GYP	PDE for c-di-GMP degradation	Promoting virulence, EPS production and DSF biogenesis	Sun et al., 2010
HGdpX3	PXO_03945	HD-GYP	PDE for c-di-GMP degradation	Promoting virulence, Inhibition of motility, EPS production and biofilm formation	Qian et al., 2017
**c-di-GMP RECEPTOR AND EFFECTOR**					
Filp	PXO_00403	Degenerate GGDEF/EAL	c-di-GMP binding, protein-protein interaction	Promoting virulence in rice, induction of hypersensitive response in non-host, and T3SS gene expression	Yang et al., 2014
PilZX1	PXO_00049	PilZ	c-di-GMP binding, protein-protein interaction	Suppressing virulence, promoting sliding motility	Yang et al., 2015
PilZX2	PXO_02374	PilZ	c-di-GMP binding, protein-protein interaction	Suppressing virulence and sliding motility	Yang et al., 2015
PilZX3	PXO_02715	Degenerate PilZ	c-di-GMP receptor-interacting protein	Interacting with Filp to co-regulate virulence expression	Yang et al., 2014
Clpxoo	PXO_04006	cNMP-/HTH-CRP	c-di-GMP binding, transcriptional regulation	Promoting virulence, swimming motility, EPS production, biofilm formation, H_2_O_2_ sensitivity, and induction of hypersensitive response in non-host	Guan et al., 2009; Li et al., 2013, 2016

GGDEF domains are differentiated into three classes: enzymatically functional domains, enzymatically functional domains linked to an EAL domain, and enzymatically non-functional domains based on the domain homology and the signature/motif conservation (Römling et al., 2013; Römling and Galperin, 2017). Enzymatically functional domains consist of the GG(D/E)EF sequence motif, including the well-conserved D/E catalytic base and other residues involved in substrate binding and coordination of one of the two divalent cations. Enzymatically non-functional domains are usually characterized by a degenerate GGDEF motif, since any mutation within the motif abolishes the catalytic activity. In addition, the inhibitory site (I-site), designated by the central signature motif RXXD, is another functional feature that characterizes the activity profile (Römling et al., 2017). However, there are different studies indicating that several DGCs with some substitutions on the first amino acid of the canonical GG(D/E)EF motif are still functional in c-di-GMP synthesis (Pérez-Mendoza et al., 2011; Hunter et al., 2014; Baena et al., 2019). Sequence alignment analysis of 11 GdpXs showed the conserved GGDEF motifs in GdpX1, 4, 5, 6, 7, and 11, the conserved I-sites in GdpX2, 3, 4, 5, 6, 7, and 8, severely degenerate GGDEF motifs and I-sites in GdpX9 and GdpX10, and a degenerate GTP binding site in GdpX9 (Figure S1). Further sequence alignment analysis of an additional 10 GEdpXs indicated the conserved GGDEF motifs only in GEdpX2 and GEdpX10, and the degenerate I-sites in most of GEdpXs (Figure S2A). These findings suggest that most GdpXs and GEdpXs might be active and inactive DGCs, respectively. Furthermore, the proteins like GdpX10 and GEdpX1 with some substitutions on the first amino acid of the GG(D/E)EF motif might be DGC active.

EAL domains are divided into three classes: catalytically active, potentially catalytically active, and catalytically inactive. EAL domains can potentially possess enzymatic activity with deviation of residues from the conserved motif. Conserved EAL motifs and critical metal cation and substrate binding sites are vital for structure and function of the protein (Römling et al., 2017). Sequence alignment analysis indicated that EAL motifs and residues were conserved in GEdpXs, except for GEdpX3 (Filp) and GEdpX9 (PXO_02944) and all of EdpXs (Figures S2B, S3). Filp was experimentally confirmed to be a c-di-GMP receptor as described below (Yang et al., 2014). GEdpX9 was identified as a putative c-di-GMP receptor to negatively regulate virulence, EPS production and biofilm formation (Li et al., 2014). These data indicate that most EdpXs and GEdpXs might be PDE active, while Filp and GEdpX9 are experimentally confirmed and putative c-di-GMP effectors, respectively.

HD-GYP domain proteins with the conserved HD and GYP residues hydrolyze both c-di-GMP and 5'-pGpG into GMP (Römling et al., 2013). Sequence alignment analysis of three HGdpXs revealed that the HD-GYP domains were well-conserved in HGdpX1-3 that were predicted as the active PDEs for c-di-GMP hydrolysis (Figure S4).

Besides these regulatory domains mentioned above, several sensory domains in GdpXs, EdpXs, GEdpXs, and HGdpXs were identified through bioinformatic analysis, including the REC, PAS(C), GAF, HAMP, Cache_1, CHASE3, Reg_prop- and Y_Y_Y, MHYT, and DUF domains (Figure 1; Table 2). These data provide information that diverse signal input domains modulate the activity of downstream GGDEF, EAL, or HD-GYP output domains by perceiving various environmental stimuli within the complicated c-di-GMP signaling network in *Xoo* (Xue et al., 2015).

**Table 2:** Bioinformatic analysis of sensory domains of GdpXs, EdpXs, GEdpXs and HGdpXs in *Xanthomonas oryzae* pv. *Oryzae*.

**Sensory domain**	**Possible function**	**Protein**
REC	Forming part of response regulator of a TCS, modulating c-di-GMP levels in response to intra- or extra-cellular signals received by their cognate histidine kinase sensor.	GdpX4, GEdpX3, GEdpX6, GEdpX9, EdpX2, HGdpX1, HGdpX2
PAS(C)	Perceiving changes of oxygen, light, redox potential, small ligands, and overall energy levels of a cell	GdpX10, GEdpX1, GEdpX3, GEdpX7, GEdpX10
GAF	Regulating c-di-GMP catalytic activity by cGMP binding	GdpX5, GdpX10, GEdpX1, GEdpX4
HAMP	Regulating phosphorylation or methylation of homodimeric receptors by ligand binding	GdpX11, GEdpX2, GEdpX8
Cache_1	Involved in small-molecule recognition of various chemotaxis receptors	GdpX6
CHASE3	Containing an extracellular sensory domain is hypothesized to recognize unknown environmental signals	GdpX5
Reg_prop- and Y_Y_Y	Forming part of the periplasmic sensor domains with unknown function	GdpX2, GdpX3
MHYT	Comprised of an N-terminal triplet tandem repeat, and each repeat contained two transmembrane helices	GEdpX5
DUF3391	Not characterized yet for its function	HGdpX3

## Identification of c-di-GMP Metabolizing-Enzymes

### GdpX1 and DgcA, Two DGCs That Similarly Regulate Virulence Factor Traits

GdpX1 (PXO_00649) encodes a well-conserved GGDEF domain standalone protein that was predicted as a putative DGC (Figure 1; Figure S1). Its regulatory function in virulence was identified by genetic and biochemical analysis (Yang et al., 2016). Deletion of *gdpX1* resulted in increased virulence, EPS production, and flagellar motility, while overexpression of GdpX1 in the wildtype led to attenuated virulence and decreased EPS synthesis. Moreover, GdpX1 suppressed the transcription of genes involved in type III secretion system (T3SS) activation, EPS synthesis, and flagellar motility. These results imply that GdpX1 is a putative DGC and suppresses bacterial virulence traits in this pathogen (Table 1) (Yang et al., 2016). Further characterization of the DGC activity of GdpX1 and intracellular c-di-GMP levels in the gene deletion mutant and overexpression strains will be helpful for understanding the regulatory mechanisms of virulence by GdpX1 in *Xoo*.

Similarly, DgcA (XOO3988) from *Xoo* strain KACC10331 encodes a well conserved GGDEF domain protein with 99.6% sequence identity to GdpX11 (PXO_04275) from PXO99^A^. The DgcA's DGC activity was first characterized in the study of a c-di-GMP riboswitch (Zhou H. et al., 2016). DgcA negatively affected bacterial pathogenicity on rice, EPS production, motility, and autoaggregation via modulating the intracellular c-di-GMP levels, demonstrating DgcA as an active DGC and major virulence regulator of in *Xoo* (Su et al., 2016). Furthermore, DgcA affected the expression of an array of genes encoding GGDEF and EAL domains, indicating an additional role of DgcA in the regulation of DGC and PDE expression (Su et al., 2016) (Table 1).

### EdpX1, an Active PDE That Promotes Virulence, EPS Production, and Biofilm Formation

EdpX1 (PXO_03877), encoding a well conserved EAL domain standalone protein, was annotated as a PDE (**Figures 1**, **3**). Genetic assays indicated that deletion of *edpX1* led to increased intracellular c-di-GMP levels, which can be restored to wildtype levels via complementation of *edpX1*. *In vitro* colorimetric assays showed EdpX1 displayed PDE activity for c-di-GMP degradation, and a point mutation of E^153A^ at the EAL motif strongly reduced its activity. Δ*edpX1* exhibited a severely impaired virulence phenotype in rice, EPS production, and biofilm formation. *In trans* expression of wildtype *edpX1*, but not *edpX1*^E153A^, was able to restore these phenotypes to near wild type levels. These observations indicate that EdpX1 functions as an active c-di-GMP-specific PDE and promotes virulence, EPS production, and biofilm formation (Table 1) (Xue et al., 2018).

### PdeR, a PDE That Is Promoted by Its Cognate HK PdeK and Interactor TriP

It was previously reported that a HK/RR pair of a TCS of Xcc, RavS (XCC1960)/RavR (XCC1958), interplayed with the DSF-mediated quorum sensing (QS), and regulated EPS and extracellular protease secretion, and their homologs were well-conserved in other close species and pathovars *Xoo*, Xoc, Xcv, and Xac (He et al., 2009; Wei et al., 2016). Low-oxygen tension is a common stress in the progress of bacterial pathogen infection or in stationary phase growth, and the PAS domain normally senses environmental signals such as oxygen, redox, and light (Girvan and Munro, 2013). RavS/RavR play a key role of in regulation of bacterial virulence in response to low-oxygen tension through the PAS domain of RavS. In addition, RavS/RavR regulates virulence gene expression through the global regulator Clp (He et al., 2009).

In *Xoo* PXO99^A^, PXO_01020, and PXO_01019 (PdeR) were the homologs of RavS and RavR, respectively. PdeR, a GGDEF/EAL/REC-domain containing protein was identified as a RR to form a pair of TCS with the cognate histidine kinase (HK) PXO_01018 (PdeK), but not PXO_01020 (Figure 1) (Yang et al., 2012). PdeK showed 95.7% identity with another HK XCC1957 adjacent to RavR in Xcc. PdeR had PDE activity for c-di-GMP hydrolysis *in vitro*, which was triggered by PdeK. Attenuated virulence in rice and less EPS secretion resulted from the deletion of *pdeR* or *pdeK*, but not *PXO_01020*. These observations suggest that PdeK/PdeR is a novel TCS involved in regulation of c-di-GMP turnover and virulence expression in *Xoo* (Table 1) (Yang et al., 2012). The differences in TCS constitution between PdeK/PdeR and RavS/RavR might be due to genetic divergence of in *Xanthomonas* species and pathovars.

**Figure 2:**
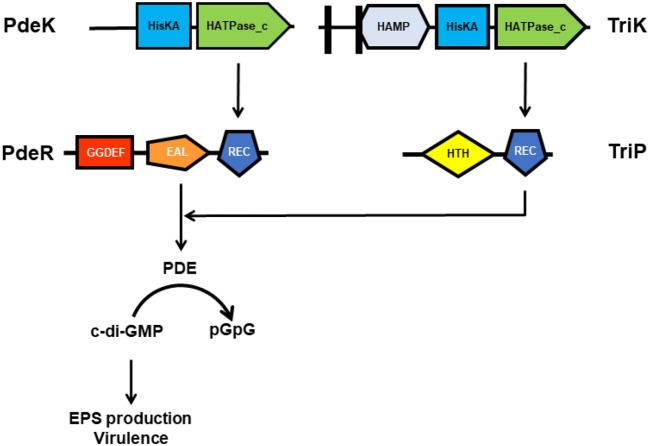
A schematic diagram showing the TriP-PdeR interaction resulting in enhanced PDE activity of PdeR in the presence of PdeK in *Xanthomonas oryzae* pv. *Oryzae*. PdeR functions as a PDE for c-di-GMP hydrolysis and forms a TCS with PdeK. TriP specifically interacts with the EAL domain of PdeR via the REC domain. The TriP-PdeR interaction significantly enhances PdeR's PDE ability for degradation of c-di-GMP into pGpG, thereby promoting EPS production and bacterial virulence in a c-di-GMP-dependent manner.

To further reveal the key elements in the PdeR-mediated c-di-GMP signaling, TriP (PXO_04421), a putative *t*ranscriptional *r*egulator *i*nteracting with *P*deR was identified and characterized. TriP was well-conserved with 100% identifies in other close *Xanthomonas* species and pathovars. The REC domain of TriP specifically interacted with the EAL domain of PdeR. TriP promoted the PDE activity of PdeR in the presence of PdeK. In-frame deletion in *triP* abolished polar localization of PdeR in the cells, significantly reduced virulence in rice, and impaired EPS production. The double mutant Δ*triP*Δ*pdeR*, like Δ*pdeR*, caused shorter lesion lengths and produced less EPS than the single mutant Δ*triP*. Cross-complementation showed *in trans* expression of *pdeR* in Δ*triP* restored its EPS production to near wildtype levels, *but not vice versa*. Accordingly, TriP is a novel regulator epistatic to PdeR in promoting virulence expression (Table 1) (Li H. Y. et al., 2018). This is the first demonstration of the REC-EAL domain interaction of two RRs and TriP, and its function in virulence regulation via PdeR-mediated c-di-GMP signaling in Xanthomonads. Though broader implications of the PdeK/PdeR/TriP/TriK signaling cascade and its conservation in *Xanthomonas* need to be further explored, a schematic diagram showing the TriP interaction with the EAL domain of PdeR via the REC domain and promotion of the PDE activity of PdeR in the presence of PdeK in *Xoo* has been presented (Figure 2). PdeR functions as a PDE for c-di-GMP hydrolysis and forms a TCS with PdeK. TriP specifically interacts with PdeR, but not PdeK. The REC and EAL domains are essential for the TriP-PdeR interaction. Such an interaction significantly affects PdeR's PDE ability to degrade c-di-GMP into pGpG and bipolar localization, thereby regulating bacterial virulence and EPS production in a c-di-GMP-dependent manner (Yang et al., 2012; Li H. Y. et al., 2018).

### HGdpX1-3, Functional PDEs That Differentially Regulate Virulence Traits

It has been generally known that DSF mediates QS signaling pathways and plays crucial roles in regulation of virulence or virulence factor production, including extracellular enzyme synthesis, EPS production, biofilm formation, and cellular dispersion in *Xanthomonas* species (Dow, 2008; Barel et al., 2015; Zhou L. et al., 2016). The sensor HK RpfC and RR RpfG constitute a TCS involved in the DSF signal perception and transduction (Slater et al., 2000; He et al., 2010). RpfC senses DSFs to activate its autokinase activity, thereby activating RpfG for PDE activity (Cai et al., 2017). RpfG contains a receiver domain for signal transduction and a HD-GYP domain for c-di-GMP degradation. In addition, RpfC regulates the expression of *hrp* genes via RpfG and the global transcriptional regulator Clp (Jiang et al., 2018). Furthermore, RpfG's regulation of downstream signaling components might be at the post-transcriptional and post-translational levels. For example, RpfG interacts with some GGDEF domain proteins and the TCS NtrBC (Andrade et al., 2006; Ryan and Dow, 2010). Proteomics analysis revealed that RpfG, RpfC, and RpfF regulated the expression of a number of new virulence factors belonging to several functional categories, including biosynthesis and intermediary metabolism, regulation, oxidative stress or antibiotic resistance, and DNA replication. Among them, the elongation factor P (XC_2359) and a putative outer membrane protein (XC_0650), both widely conserved in bacteria, were characterized as the novel virulence factors regulated by the RpfC/RpfG/RpfF system at post-transcriptional but not transcriptional level (O'Connell et al., 2013). RpfG was also identified as a global regulator controlling EPS production that contribute to biofilm formation and bacterial virulence on rice in Xoc (Zhang Y. et al., 2013).

The RpfC/RpfG-mediated DSF/c-di-GMP signaling pathways and their regulation of virulence factor production was earlier shown to be well-conserved in Xcc and *Xoo* (He et al., 2010). HGdpX1 (PXO_00070, RpfGxoo), a Xcc RpfG homolog, was identified to promote virulence in rice and EPS production (Figure 1; Figure S4) (Sun et al., 2010). The TCS RpfCxoo/RpfGxoo was reported to negatively regulate the expression of *rpfB* that played an important role in DSF signal turnover via Clp (Wang X. Y. et al., 2016). In addition, two other HD-GYP domain proteins HGdpX2 (PXO_00476) and HGdpX3 (PXO_03945) have been characterized to degrade c-di-GMP and promote virulence in *Xoo* (Figure 1; Figure S4) (Qian et al., 2017). These observations imply that HGdpX1-3 might be active PDEs, and differentially regulate bacterial virulence traits (Table 1).

In Xcc, PcrR forms a novel TCS with its cognate HK PcrK that specifically binds the plant cytokinin 2-isopentenyladenine (2iP) via its CHASE domain. Through a four-step phosphorelay, 2iP binding results in decreased autokinase activity of PcrK and phosphorylation level of PcrR, thereby activating PcrR's PDE activity for c-di-GMP degradation. The TCS PcrK/PcrR affects bacterial tolerance to oxidative stress through regulating the gene transcription. These findings reveal an inter-kingdom signaling by which Xcc intercepts a plant derived hormone signal to promote adaptation to oxidative stress (Wang et al., 2017). Furthermore, PcrK and PcrR show 81.4% and 84.91% sequence identities with PXO_00475 and PXO_00476 (HGdpX2) of *Xoo*, and 82,7% and 85.76% identities with XOC_1985 and XOC_1984 of Xoc, respectively, indicating this TCS is more widely conserved in other Xanthomonads.

## Characterization of c-di-GMP Receptors and Effectors

### Filp, a Degenerate GGDEF/EAL Domain Protein That Regulates Virulence Expression With Its Interactor PilZX3

FimX was primarily characterized as a PDE regulating twitching motility in *Pseudomonas aeruginosa* (Huang et al., 2003; Kazmierczak et al., 2006). Subsequent studies revealed that FimX functioned as a receptor for c-di-GMP binding via the degenerate GGDEF/EAL domains (Navarro et al., 2009). The FimX-like proteins in Xac were also characterized to regulate bacterial type IV pilus (T4P) biogenesis through interacting with a degenerate PilZ domain protein to form a FimX-c-di-GMP-PilZ complex; And a novel mechanism underlying the regulation of T4P biogenesis by this complex via binding to an ATPase PilB required for T4P polymerization has been proposed in Xac (Guzzo et al., 2009, 2013). This is the first evidence of direct interactions of FimX and PilZ orthologs with the TP4 machinery.

In *Xoo*, Filp (PXO_00403) was identified as a FimX-like, degenerate GGDEF/EAL domain hybrid protein, which showed 96.8 and 93.8% protein sequence identifies to FimXXac2398 and XccFimX, respectively (Figure 1; Figure S2) (Yang et al., 2014). Filp functions as a c-di-GMP binding protein through its EAL domain *in vitro*. Filp regulated virulence in rice, in which the REC, PAS, and EAL domains, but not the GGDEF domain, were required for full activity. Notably, Filp specifically interacted with a degenerate PilZ domain protein PilZX3 (PXO_02715). Deletion of *pilZX3* led to the same changes in bacterial virulence and T3SS gene expression as the Δ*filp* mutant. This indicates that Filp is a novel c-di-GMP receptor to co-regulate bacterial virulence with its interactor PilZX3 (Table 1) (Yang et al., 2014). XOC_2102, a Filp homolog in Xoc, showed same ability for c-di-GMP binding (Wei et al., 2016). Taken together, the regulation of bacterial motility and virulence mediated by FimX-like proteins might be well-conserved in *P. aeruginosa* and *Xanthomonas* spp.

Deciphering the complexity of c-di-GMP signaling is of importance for a comprehensive understanding of molecular mechanisms underlying bacterial virulence expression and regulation (Römling et al., 2013). To elucidate the regulatory mechanisms of virulence by Filp/PilZX3, isobaric tags for relative and absolute quantitation (iTRAQ) analysis of Δ*filp*, Δ*pilZX3*, and Δ*filp*Δ*pilZX3*, compared with the wildtype strain was performed. A subset of differentially-expressed proteins including c-di-GMP metabolizing enzymes, HKs, and TonB receptors were identified, in which two PDEs, VieAxoo and HGdpX2, significantly affected bacterial virulence in a Filp/PilZX3-dependent manner (Table 1) (Liang et al., 2011). This provides evidence of the existence of novel feedback regulatory circuits comprising Filp, PilZX3, and VieAxoo or HGdpX2 in c-di-GMP signaling networks of *Xoo*.

### PilZX1-4 Function Differentially in c-di-GMP Binding and Regulation of Virulence

PilZ domain containing proteins have been known as one of the major types of c-di-GMP receptors/effectors in many bacteria (Chou and Galperin, 2016). PliZ domain proteins are widely conserved in the closely-related *Xanthomonas* spp. Four PilZ domain proteins were previously reported to be involved in the regulation of virulence and related traits in Xcc. Among them, XC0965, XC2249, and XC3221 promoted the virulence in Chinese radish, XC2249 and XC3221 enhanced the motility, and XC2249 and XC0965 regulated extracellular enzyme production of McCarthy et al. (2008).

In *Xoo*, PilZX1-4, which constitute 4 homologous proteins PXO_00049, PXO_02374, PXO_02715, and PXO_00997/PXO_06169, were identified through bioinformatics analysis. Both PilZX1-2 possessed the conserved PilZ domains, while PilZX3-4 harbored the degenerate ones (Figure S5). PilZX1-2 directly bound to c-di-GMP with high affinity, and negatively affected bacterial virulence and sliding motility, while PilZX3 functioned as a specific interactor of the c-di-GMP receptor Filp, and positively regulated bacterial virulence (Table 1) (Yang et al., 2014, 2015). Furthermore, PilZX1-2 displayed multisite subcellular localizations, whereas PilZX3 showed non-polar distributions in the cells (Yang et al., 2015).

### Clpxoo, a c-di-GMP Receptor and Transcription Regulator

Since CRP was first identified to bind with cAMP in *Escherichia coli*, it has been extensively studied as a global regulator to regulate carbon source metabolism in bacteria and other signaling pathways, through controlling the transcription of downstream genes (Weber and Steitz, 1987; Zheng et al., 2004; Almagro et al., 2018). A *C*RP-like *p*rotein Clp was identified as a c-di-GMP receptor, and c-di-GMP allosterically inhibits the DNA-binding activity of transcription factor Clp in Xac (Leduc and Roberts, 2009). The role of Clp was confirmed as a c-di-GMP effector, whose binding to the promoter of the endoglucanase gene *engXCA*, was inhibited by c-di-GMP in Xcc (Chin et al., 2010; Tao et al., 2010). Clp regulated the transcription of a number of genes revealed by DNA microarray analysis (He et al., 2007). Structural and functional analysis of Clp showed key c-di-GMP binding sites D70, R154, R166 and D170, and the target promoter binding sites E99, R150, R195, D162, and V165 (Chin et al., 2010; Tao et al., 2010). In *Xoo*, Clpxoo (PXO_04006) containing the cNMP-/HTH-DNA binding domain was identified as a c-di-GMP receptor and transcription factor (Guan et al., 2009). Structurally, Clpxoo may substantially change upon interacting with c-di-GMP,resulting in inhibition of binding to *engAxoo*-p (Li et al., 2013). According to the site-directed mutagenesis of Clp in Xcc, Clpxoo^D70A^, Clpxoo^E99S^ and Clpxoo^R166A^, site 70 of aspartic acid and 99 of glutamic acid were identified to be two key residues for Clpxoo's binding to c-di-GMP (Li et al., 2016). Furthermore, Clpxoo positively regulated virulence, EPS production, biofilm formation, and resistance to hydrogen peroxide (H_2_O_2_) (Guan et al., 2009). These findings manifest that Clpxoo is a c-di-GMP effector to regulate bacterial virulence phenotypes at the transcriptional levels in *Xoo*.

## Summary and Future Issues

### Implication of c-di-GMP Signaling Pathways in Regulation of Virulence in *Xoo*

**Figure 3:**
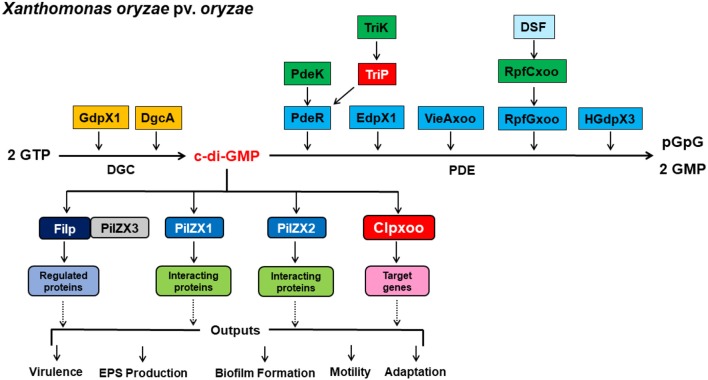
A proposal model outlining c-di-GMP signaling regulation of virulence expression in *Xanthomonas oryzae* pv. *Oryzae*. c-di-GMP is a ubiquitous, bacterial second messenger that regulates phenotypes including virulence, EPS production, biofilm formation, motility, and adaptation. c-di-GMP is produced by DGCs GdpX1 and DgcA, and degraded by PDEs PdeR, EdpX1, VieAxoo, RpfGxoo, and HGdpX3. PdeK/PdeR, TriK/TriP, and RpfCxoo/RpfGxoo are 3 pairs of the histidine kinase/response regulator of two component systems (TCSs). c-di-GMP is sensed by receptor proteins Filp, PilZX1, PilZX2, and Clpxoo. Particularly, Filp interacts with PilZX3 for c-di-GMP binding and downstream regulation. Signal receptor and effector proteins then interact with the downstream target proteins or genes to affect a particular cellular function and phenotype, including bacterial virulence, EPS production, biofilm formation, motility, and adaptation at the transcription, post-translation and protein-protein interaction levels.

Resolving the highly complex pathways of c-di-GMP signaling is of utmost importance for a comprehensive understanding of the molecular mechanisms underlying the regulation of virulence expression in plant-pathogenic bacteria, including *Xanthomonas* spp. Recent studies have uncovered several components involved in c-di-GMP signaling pathways and led to significant gains in our knowledge and understanding of c-di-GMP regulatory circuits of virulence-related traits in *Xoo*. Here we proposed model outlining the identified c-di-GMP signaling components and their regulation of virulence in *Xoo* (Figure 3). An array of GGDEF, EAL, and HD-GYP domain proteins encoded in the genome were identified as DGCs and PDEs for c-di-GMP synthesis and degradation. PdeR, EdpX1, RpfGxoo, VieAxoo, and HGdpX3 functioned as PDEs to degrade c-di-GMP, and promotes EPS production, biofilm formation, and virulence. In contrast, GdpX1 and DgcA acted as DGCs and controlled EPS production and virulence in a manner opposite to PdeR, EdpX1, RpfGxoo, VieAxoo and HGdpX3. This regulation scheme is consistent with the general theory that low c-di-GMP levels promote virulence factor production, while high c-di-GMP levels inhibit virulence factor production in pathogenic bacteria.

### Functional Diversification of GGDEF/EAL/HD-GYP Domains

GGDEF, EAL, and HD-GYP domain proteins are widely distributed in bacteria, and some of them have been characterized in *Xanthomonas* spp (Schirmer and Jenal, 2009; Yang et al., 2012; Wei et al., 2016). For example, the roles of 11 GGDEF-EAL domain proteins were revealed in Xoc (Wei et al., 2016). Among them, XOC_2335 and XOC_2393 enhanced bacterial swimming motility, while XOC_2102, XOC_2393, and XOC_4190 inhibited sliding motility. In addition, XOC_2335, XOC_2393, and XOC_4190 were identified to be involved in regulating virulence to rice. Notably, XOC_2335 was a homolog of GEdpX5 unidentified in *Xoo*, while XOC_2393, XOC_2102, and XOC_4190 were the homologs of PdeR, Filp, and GEdpX9 that were characterized in *Xoo*, respectively. XOC_2102 showed same ability for c-di-GMP binding as Filp did, and XOC_4190 and GEdpX9 might function as the novel c-di-GMP receptors in these two pathvars. Furthermore, dynamic interactions between RpfG and two GGDEF domain proteins, XC_0249 and XC_0420, were characterized to be dependent on DSF signaling in Xcc (Ryan and Dow, 2010; Ryan et al., 2015). There might exist a complicated protein-protein interaction network between the GGDEF, EAL, and HD-GYP domain proteins in *Xoo* (Guo et al., 2013). Accordingly, these GGDEF/EAL/HD-GYP domain proteins may function not only as c-di-GMP metabolizing enzymes or receptors, but also act as elements within interacting networks to regulate bacterial processes. Further exploration of the interactome of GGDEF, EAL, and HD-GYP domain proteins will helpful to uncover the c-di-GMP signaling protein network and will hopefully allow a comprehensive understanding of the regulatory mechanisms of bacterial virulence at the post-translational and protein-protein interaction levels.

### Further Exploration of Novel c-di-GMP Receptor Domains

Several c-di-GMP receptors have been characterized in *Xoo*, including one degenerate GGDEF/EAL domain protein, 4 PilZ domain proteins, and one transcriptional regulator (Yang et al., 2017). Since the number of DCGs and PDEs encoded in the genome is far larger than that of known c-di-GMP receptors, it is likely that more receptors remain to be explored in this pathogen. An array of new c-di-GMP receptors and effectors has been recently identified in Xanthomonads and other bacteria. One example is YajQ (XC_3703), a PNPase from Xcc, and XOC_4190 from Xoc, which was well-conserved in *Xanthomonas* spp (An et al., 2014; Zhao et al., 2016). YajQ showed 96% identity with PXO_03091, and XOC_4190 displayed 97.4% similarity with GEdpX9, suggesting that PXO_03091 and GEdpX9 might be two new c-di-GMP receptors in *Xoo*. In addition, a Differential Radial Capillary Action of Ligand Assay (DRaCALA) screen identified the c-di-GMP receptor MshE, an ATPase associated with the mannose sensitive hemagglutinin (MSHA) T4P in *Vibrio cholerae*, meanwhile an ATPase of type II secretion system (T2SS) in *Pseudomonas aeruginosa*, the homolog of MshE was also identified as a signal receptor, indicating both T4P and T2SS are regulated by c-di-GMP signaling (Roelofs et al., 2015). The N-terminal of MshE (MshEN) was characterized to be the c-di-GMP receptor domain with two 24-residue motifs [RLGxx(L)(V/I)xxG(I/F)(L/V)xxxxLxxxLxxQ] that were linked by a 5-residue spacer to form a complete 53-residue-long domain (Wang Y. C. et al., 2016). This domain was also found present in XpsE or GpsE, a T2SS protein in Xcc (Shiue et al., 2006), while PXO_02675 in *Xoo* is a homolog of XpsE with 94.26% sequence identity. An affinity capture screen for c-di-GMP binding proteins was performed and flagella export AAA+ ATPase FliI was identified to be c-di-GMP receptor in *P. fluorescens*. ATPases HrcN of T3SS in *P. syringae* pv. *tomato* and ClpB2 of T4SS in *P. fluorescens*, the homologs of FliI were identified as c-di-GMP receptors (Trampari et al., 2015). Therefore, ATPase is a novel class of c-di-GMP receptor. Among 62 proteins annotated as ATPases in *Xoo*, PXO_02675 (a T2SS protein), PXO_03396 (a T3SS protein HrcN), and PXO_06188 (a flagellar protein FliI) might be the new c-di-GMP signal receptors. Moreover, diverse proteins and riboswitches were identified as novel c-di-GMP effectors in other pathogenic bacteria, for instance, FleQ, a transcription regulator from *P. aeruginosa*, and SgmT, a HK from *Myxococcus xanthus* (Kulshina et al., 2009; Baraquet et al., 2012; Petters et al., 2012; Tang et al., 2016; Weinberg et al., 2017). FleQ's homologs widely existed in Xanthomonads, which were the major transcriptional activators of expression of flagellar genes in Xcc and *Xoo* (Tian et al., 2015; Bae et al., 2018). Furthermore, Many HKs were extensively characterized for their biological functions in Xcc and *Xoo* (Qian et al., 2008). Therefore, identification of these novel c-di-GMP receptors in other bacteria would be informative for further exploration of the unknown signal receptors and effectors in *Xoo*.

### Multiple Regulatory Mechanisms Mediated by c-di-GMP Receptors

It has been generally recognized that c-di-GMP receptors exert their regulatory function at the transcriptional and post-transcriptional levels. For example, Clp of Xcc and Clpxoo of *Xoo* function as transcription factors to affect bacterial functions through direct binding of the target gene promoters to control transcription. Recent studies showed that c-di-GMP receptors including Filp, FimXxac, and YajQ transmitted communication via protein-protein interaction in *Xanthomonas* spp., and intracellular c-di-GMP concentrations significantly affected the interactions between the signal receptors and their downstream proteins (Guzzo et al., 2009, 2013; An et al., 2014; Zhao et al., 2016). Furthermore, a novel mechanism underlying the Clp-LchP complex formation that results in increased PDE activity of LchP has been recently demonstrated in *Lysobacter* (Xu et al., 2018). Thus, deciphering the signaling networks formed by c-di-GMP receptors and its interactors, particularly between PDEs and DGCs, would be helpful to comprehensively understand the molecular mechanism underpinning the c-di-GMP receptor-mediated regulation in pathogenic bacteria.

Taken together, more investigation is required for deciphering the molecular mechanisms of c-di-GMP signaling during bacterial pathogenesis and adaptation to environmental stresses. In order to further our understanding, the next 5 steps must be taken: (1) characterize the functions of the remaining GGDEF, EAL, and HD-GYP domain proteins, (2) determine the environmental cues that affect the DGC and PDE activities and intracellular c-di-GMP levels through the sensory input domains, (3) characterize the c-di-GMP receptor-mediated regulatory pathways via the transcription and post-translation mechanisms, (4) assess c-di-GMP regulation of bacterial type III secretion system (T3SS), and (5) uncover the specificity in c-di-GMP signaling through protein-protein interaction mechanisms.

## Author Contributions

FY and DX performed experiments. FY, DX, FT, WH, C-HY, and CH analyzed data and wrote the manuscript.

### Conflict of Interest Statement

The authors declare that the research was conducted in the absence of any commercial or financial relationships that could be construed as a potential conflict of interest.
