# Supplementary material for: Identification of c-di-GMP Signaling Components in Xanthomonas oryzae and Their Orthologs in Xanthomonads Involved in Regulation of Bacterial Virulence Expression
Source: Front Microbiol. 2019 Jul 11;10:1402. doi: 10.3389/fmicb.2019.01402 (PMC6637768; doi:10.3389/fmicb.2019.01402)
Supplement: Supplementary file 1 [file Presentation_1.pdf]

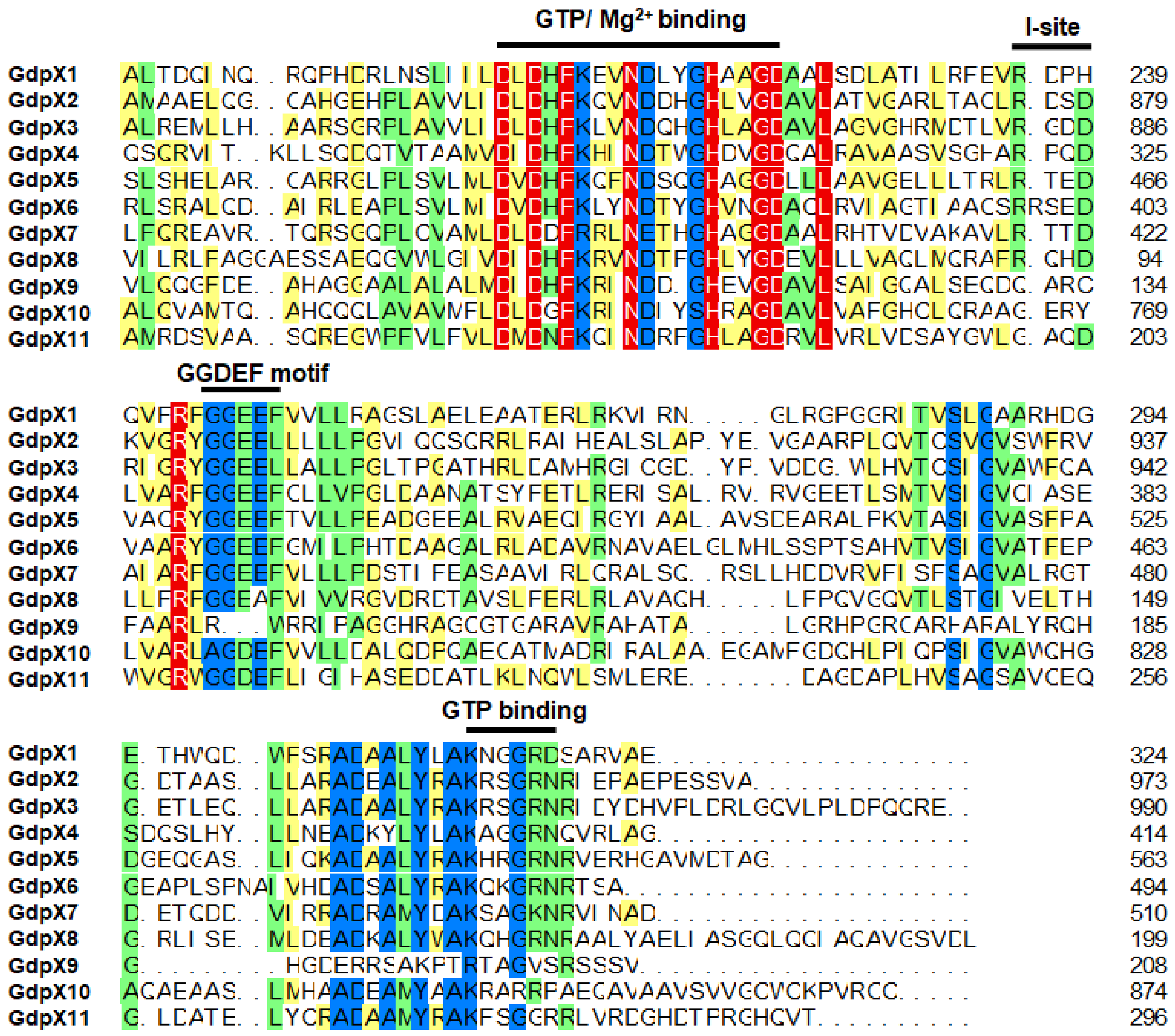

Fig. S1 Sequence alignment of the GGDEF standalone proteins GdpXs

Multiple sequence alignments were carried out by using DNAMAN software (version 6.0, Lynnon BioSoft, Canada). The amino acids highlighted with red, blue, green, and yellow mean 100%, ≥75%, ≥50% and ≥33% identities, respectively. Black lines showed the corresponding sites of GTP/Mg2+ binding, I-site, GGDEF motif and GTP binding sites, respectively.

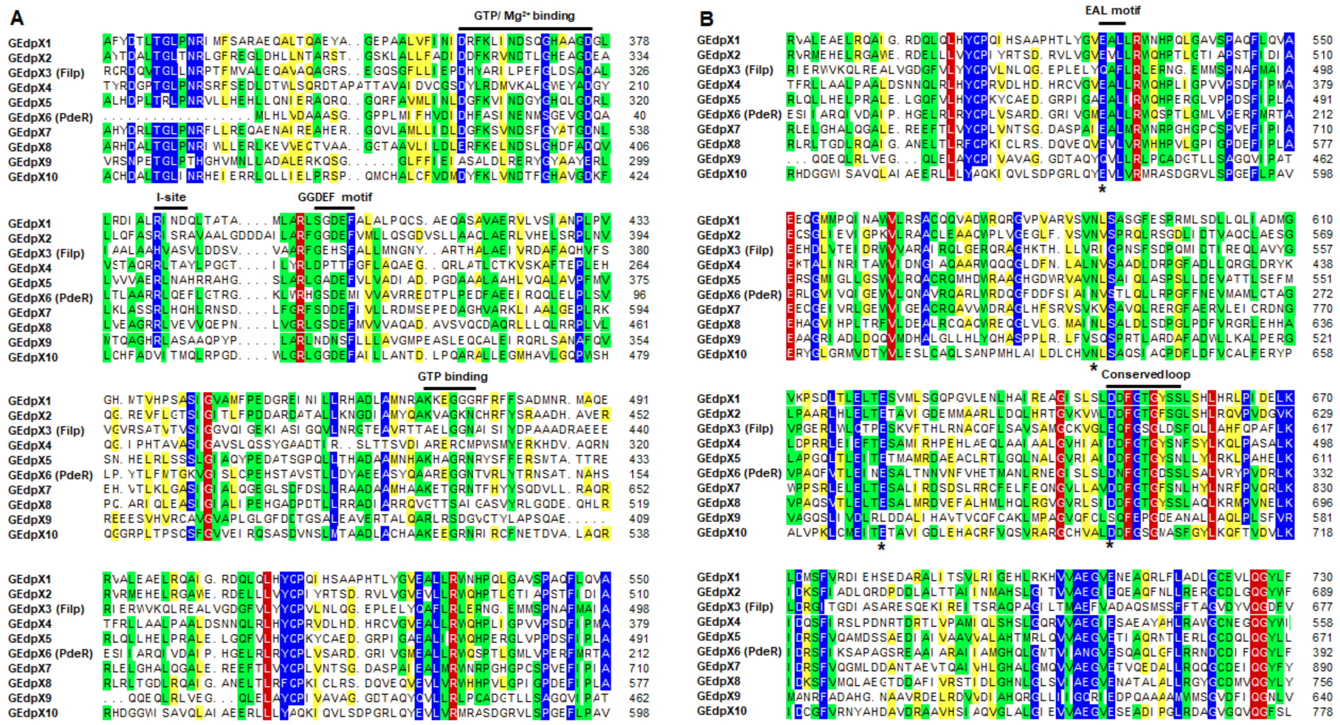

Fig. S2 Sequence alignment of the GGDEF and EAL hybrid proteins GEdpXs

Multiple sequence alignments were carried out by using DNAMAN software (version 6.0, Lynnon BioSoft, Canada). The amino acids highlighted with red, blue, green, and yellow mean 100%, ≥75%, ≥50% and ≥33% identities, respectively. **(A) The alignment of GGDEF domain**. Black lines showed the corresponding sites of GTP/Mg2+ binding, I-site, GGDEF motif, and GTP binding sites, respectively. **(B) The alignment of EAL domain**. Asterisks showed the critical metal cation and substrate binding sites. Black lines showed EAL and Loop 6 residues, respectively.

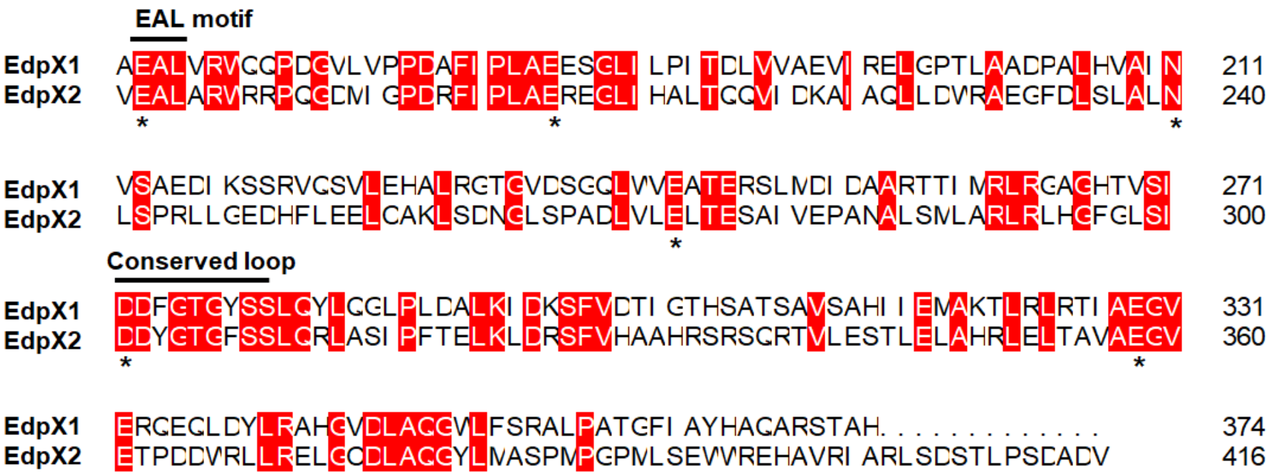

Fig. S3 Sequence alignment of the EAL standalone proteins EdpXs

Multiple sequence alignments were carried out by using DNAMAN software (version 6.0, Lynnon BioSoft, Canada). The amino acids highlighted with red means 100% identity. Asterisks showed the critical metal cation and substrate binding sites. Black lines showed EAL and Loop 6 residues, respectively.

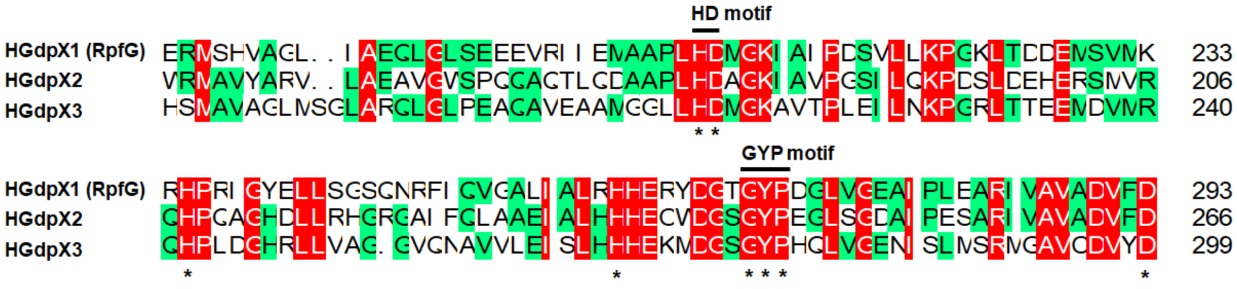

Fig. S4 Sequence alignment of the HD-GYP proteins HGdpXs

Multiple sequence alignments were carried out by using DNAMAN software (version 6.0, Lynnon BioSoft, Canada). The amino acids highlighted with red and green mean 100% and ≥50% identities, respectively. Asterisks showed the critical metal cation and substrate binding sites. Black lines showed HD and GYP motif, respectively.

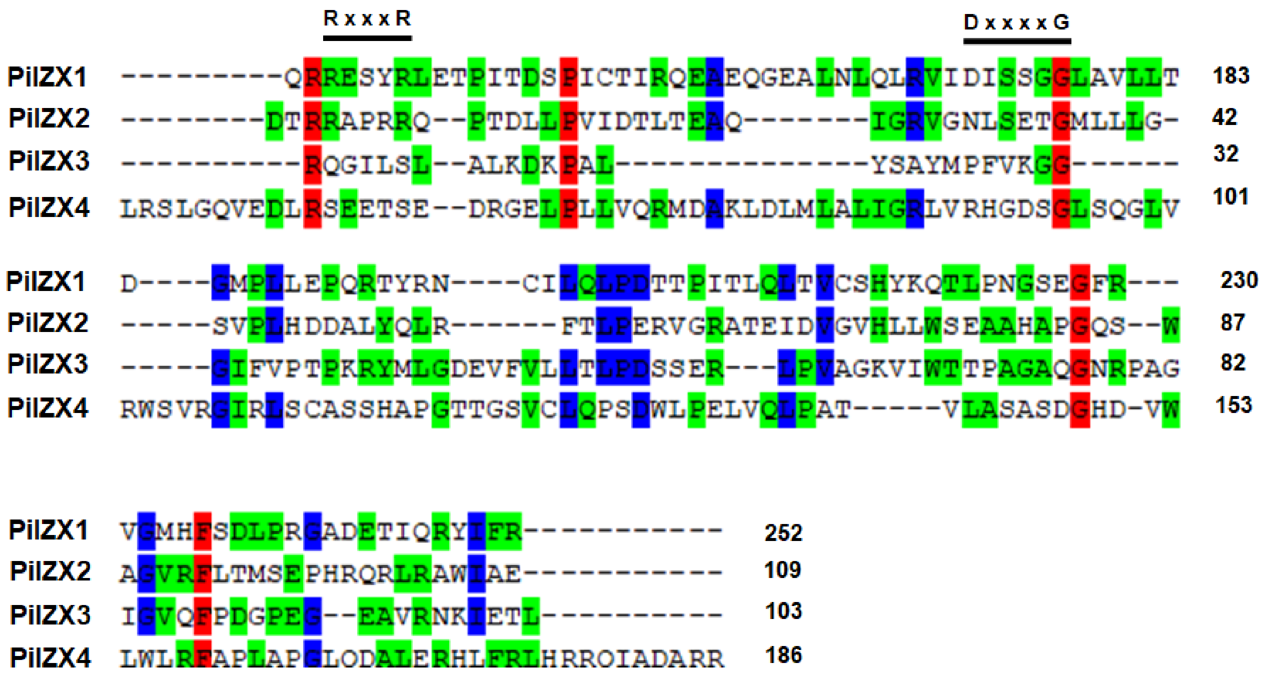

Fig. S5 Sequence alignment of the PilZ domain proteins PilZXs

Multiple sequence alignments were carried out by MEGA Muscle software analysis (version 7.0.21) and edited by using Genedoc software. The amino acids highlighted with red, blue, and green mean 100%, 75%, and 50% identities, respectively. Black lines showed the conserved motifs that were involved in c-di-GMP binding in the PilZ domain.
